# EFFECT OF HOME CARE NURSES’ REMOTE CONSULTATION WITH ADVANCED NURSES ON PATIENT SYMPTOMS: A FEASIBILITY STUDY

**DOI:** 10.1093/geroni/igae098.2794

**Published:** 2024-12-31

**Authors:** Maiko Noguchi-Watanabe, Reiko Yamahana, Junko Sunada

**Affiliations:** Tokyo Medical and Dental University, Tokyo, Japan; Musashino University, Nishitokyo, Tokyo, Japan; IMSUT Hospital, University of Tokyo, Tokyo, Japan

## Abstract

Homecare nurses provide care for patients with a wide variety of illnesses, which requires a broad and comprehensive knowledge base. Simultaneously, symptom management requiring more specialized knowledge is required, and remote consultation with advanced nurses is expected. This study aimed to evaluate the effect of remote consultation by homecare nurses with advanced nurses on patient symptoms. Participants were homecare nurses in Japan. A remote symptom management consultation regarding specific cases was sought by ten homecare nurses. Consultations were provided thrice per case using Zoom at approximately one-month intervals by three advanced nurses: two wound, ostomy, and continence (WOC) care nurses and one cancer nurse. The research team selected the advanced nurse according to the case and coordinated the consultation schedule between advanced and homecare nurses. Before each consultation, we obtained responses from homecare nurses via a webform regarding patient who wanted to consult, their symptoms, and changes in symptoms since the previous consultation (worsening/maintenance/improvement). The study was approved by the Ethics Review Committee of the Tokyo Medical and Dental University. The WOC nurses provided consultations on six cases related to pressure ulcers, two, to stomas, and one, to nephrostomy management, while an advanced nurse specializing in cancer nursing provided consultation on one case related to decision-making support. Consultations took place three times per participant. Nine cases (90%) improved their symptoms, and 1 case (10%) remained unchanged before the consultation; It has been shown that homecare nurses who receive consultations from advanced nurses, even remotely, is effective in alleviating patients’ symptoms.

